# Apple cultivar-specific pectin properties impact apple puree functionality

**DOI:** 10.1016/j.fochx.2025.103289

**Published:** 2025-11-13

**Authors:** Dazhi Liu, Jinfeng Bi, Xuan Liu, Jianing Liu, Henk.A. Schols

**Affiliations:** a Institute of Food Science and Technology, Chinese Academy of Agricultural Sciences (CAAS), Key Laboratory of Agro-Products Processing, Ministry of Agriculture and Rural Affairs, Beijing 100193, China; b Laboratory of Food Chemistry, Wageningen University & Research, Bornse Weilanden 9, 6708, WG, Wageningen, the Netherlands; c Institute of Western Agriculture, Chinese Academy of Agricultural Sciences, Changji 831100, China; d Department of Food Science and Nutrition, The Hong Kong Polytechnic University, Hong Kong SAR, China

**Keywords:** Apple cultivars, Apple puree, Pectin structure

## Abstract

Thirteen apple cultivars were investigated. to link quality parameters of purees with structural characteristics of pectins. The purees differed especially in particle size and viscosity. Pectins were extracted from the alcohol insoluble solids (AIS) and fractionated into water- and chelating-agent soluble solids (WSS & ChSS). Sugar composition differences were minimal, while esterification varied considerably. For AIS and WSS, the DM were 57–66 % and 64–79 % respectively. ChSS were categorized as either low or high-methyl-esterified (29 % to 44 % and 52 % to 78 %). Enzymatic fingerprinting demonstrated the different methyl-ester distribution within the pectin backbones. PCA demonstrated correlations between pectin structure and puree properties, illustrating that pectin characteristics significantly influence the texture of apple-based products. Sugar composition of the pectin fractions correlated with puree's rheological behaviour, while the particle size (D[4,3] 235.2–387.7 μm) was associated with the methyl-ester distribution. This study offers insights for utilizing plant polysaccharide diversity in food design.

## Highlights

• Dry matter and pulp content drive puree viscosity and stability.
• Pectin structure defines viscosity via linear chains and ester patterns.
• Pectin methyl-ester patterns regulate crosslinking, designing puree texture.

## 1 Introduction

In the apple processing industry, apples are mainly squeezed to juice, while the pomace remaining rich in cell wall polysaccharides is left behind (Shalini & Gupta, 2010). For another important utilisation of apples, apple puree, the whole edible part of the fruit is directly grinded to yield the puree (Buergy et al., 2020). These purees have become increasingly popular due to their high nutritional value meeting the demands of health-conscious consumers (Ravichandran et al., 2023).

Apple purees are considered a good source of dietary fibres because of the high concentration of cell wall polysaccharides in this consumer product (Bondonno et al., 2017). Apple purees are suitable for elderly and infants because of their soft and fluid textures and can also be used as an ingredient for plant-based products (Rusu et al., 2020). The texture of fruit puree is significantly influenced by the content and properties of the cell wall material like pectin, hemicellulos, cellulose and protein, as well as the processing conditions like thermal or non-thermal processing. The purees texture can be assessed using quality indices such as particle size distribution and viscosity (Buergy et al., 2020).

Among the plant cell wall materials, pectin polysaccharides are the most important components, which can contribute to maintaining the strength of the cell wall and determinant for defining the puree's texture (Buergy et al., 2020; Mohnen, 2008). The homogalacturonan (HG) structural element consisting of a linear chain of α 1–4 linked galacturonic acids (GalAs) represent 70 % of the pectic material and is considered as the backbone of the pectin molecule (Mohnen, 2008). Galacturonic acids within the HG backbone can be methyl-esterified at the C-6 position, and the percentage of methyl-esterified GalA to the total GalAs over the HG is defined as the degree of methyl-esterification (DM) (Levigne et al., 2002; Mohnen, 2008). Methyl-esters can be either randomly or blockwise distributed over the HG backbone, and non-esterified GalA can form blocks which affect the calcium ions binding ability of pectin (Liu et al., 2023; Voragen et al., 2009). Pectins also consist of rhamnogalacturonan I (RG-I), rhamnogalacturonan II (RG-II) and xylogalacturonan (XG) structural elements (Willats et al., 2001). Neutral sugar side chains of RG-I are composed of galactose and/or arabinose and are attached to the rhamnose through the O-2 or O-3 position (Kaya et al., 2014). Pectin's gelling, thickening and emulsifying properties are believed to depend on pectin structure characteristics such as sugar composition and content, the degree of methyl-esterification and the molecular size, where the pectin molecules side chain length and the level of substitutes will hinder the cross-linking abilities with other components or block the water binding ability of the pectin (Sila et al., 2009; Voragen et al., 2009; Willats et al., 2006). Beyond DM, the distribution pattern of methyl-esterification over the backbone is suggested to play a role in pectin functionality since different distribution patterns will bring varying cross-linking abilities and consequent influence textural properties of pectin-containing food (Sila et al., 2009; Ström et al., 2007).

The structure of pectins varies notably based on the source and developmental stage of plant tissue, particularly concerning the quantity, and location of methyl-esters within the pectin molecule (Mohnen, 2008; Sila et al., 2009). Characterizing pectin structure is meaningful in comprehending the quality of apple puree (Buergy et al., 2020; Buergy, Rolland-Sabaté, Leca, Falourd, et al., 2021). Many studies to date have focussed on the effect of added pectin as an ingredient on puree quality and processing methods on pectin structure, as well as on the basic structural characteristics of pectins, including sugar compositions, molecular weight, and DM (Christiaens et al., 2012; Salinas et al., 2019). However, the research on how the structural characteristics of fruit's native pectins, particularly the distribution of methyl-esters, affect the puree's qualities are limited. To characterise the methyl-ester distribution pattern of the pectin, an enzymatic fingerprinting approach has been proposed to obtain detailed information on the methyl-ester patterns of pectins from various apples in our previous work (Jermendi et al., 2022; Liu et al., 2023).

In a previous study, we confirmed that the fine structure of native apple pectin from various cultivars differ significantly can influenced the physical properties of apple juice (Liu et al., 2025). Thus, we hypothesised that apple varieties with variable pectin structures, particular different in blockwise methyl-ester distribution patterns, will result in different physical properties of the apple puree, like viscosity and particle size, etc. In this study, we explored the structural characteristics of pectins from 13 different apple cultivars and their effect on the properties of apple products. The outcomes of this research can contribute to comprehending variations in puree texture and quality among various apple cultivars.

## 2 Material and methods

### 2.1 Plant materials

#### 2.1.1 Raw apples

Thirteen different apple cultivars were collected on early October 2020 from an orchard in Shaanxi province, China. Apple cultivars included Golden Delicious, Hua Pi, Pink Lady, Shen Fu, Hua Niu, Cameo, Fuji, Rui Xue, Shou Erhong, Granny Smith, Ji Guan, Rui Yang and Wang Lin were selected as materials and all the apple cultivars were of similar ripeness degree. Fresh apples without storage were washed and cut into cubes. The cubes were separated into two portions, one for pectin characterization and one for puree processing.

The dry matter of apples was estimated according to AOAC (2000), with slight modification(Horwitz & Latimer, 2000). Apples were sliced and the weight (*M*_*0*_) was exactly recorded. Pieces were placed at 105 °C in an oven for 24 h until a constant weight was achieved, and dried apple pieces were weighed again (*M*_*1*_). The dry matter (*M*_*d*_) in apple tissue can be expressed as follows:

(1) $$M_{d}=\frac{M_{1}}{M_{0}}\times 100\%$$

#### 2.1.2 Puree preparation

Apple cubes of individual cultivars for puree processing were directly blended into purees with a lab-scale blender, and ascorbic acid (0.5 ‰) was added to limit oxidation. The apples were ground at 5000 rpm for 3 min at room temperature to obtain purees. Purees were stored in well-sealed ziplock bags at 4 °C for further analysis including rheology, particle size distribution, Zeta-potential, turbidity and stability.

### 2.2 Puree physicochemical characterization

#### 2.2.1 Pulp content

The pulp content or solid ratio was measured by centrifugation of puree at 5000 *g* for 2 h. The pulp content was expressed as the ratio of wet weight (*W*_*p*_) and the initial sample weight (*W*_*i*_) (Leverrier et al., 2016).

(2) $$\mathrm{Pulp ratio}=\frac{W_{p}}{W_{i}}\times 100$$

#### 2.2.2 Total soluble solids of apple puree

The total soluble solids (TSS) content of each juice sample was determined in degrees Brix using a digital refractometer (Shanghai Electronics and Analytical Instrument, China). Titratable acidity (TA) was assessed using a pH Stat Titration Unit (907 Titrando, Metrohm Ltd., Switzerland) with a standardized 0.1 M NaOH solution, and results were reported as malic acid equivalents.

#### 2.2.3 Turbidity and relative turbidity

The turbidity of apple purees refers to the degree of opaqueness caused by suspended solids, measured in nephelometric turbidity units (NTU). The purees were initially diluted 10 times, and NTU was assessed using a portable turbidimeter (Model 2100P), following the method outlined by Mollov et al. (2006). The turbidity of the apple puree was calculated by multiplying the measured values by 10. Relative turbidity (*T*_*rel*_) was determined using the formula:

(3) $$T_{\mathrm{rel}}=\frac{T_{1}}{T_{0}}*100$$

where *T*_*0*_  is the turbidity of the apple purees before centrifugation, and *T*_*1*_  is the turbidity of the supernatant obtained after centrifuging the apple puree at 4200 g for 15 min at 20 °C.

#### 2.2.4 Zeta-potential and total soluble solids

A Zetasizer Nano-ZS (Malvern Instruments, Malvern Worcestershire, UK) was used to determine particle charge. The Zeta-potential was measured in water and at pH 4 (acetate buffer, 5 mM). Before analysis, each sample was diluted 20 times.

#### 2.2.5 Particle size distribution

A laser particle size analyzer was used to determine the particle size distributions of apple purees (model S3500, Microtrac, USA). The median particle diameter (D50), volume-based diameter (D[4,3]), and area-based diameter (D[3,2]) were all measured and expressed in μm.

#### 2.2.6 Viscosity and viscoelasticity

Rheological measurements were determined with a Physica MCR301 rheometer (Anton Paar, Austria using parallel plate geometry (40-mm diameter) with a gap size of 1 mm.

Viscosity curves (shear stress versus shear rate) were recorded. Different models were used to fit the rheological curves (Basu et al., 2013), and the results indicated that the Herschel-Bulkley model (R^2^ > 0.96) was the best fit,

(4) $$\tau =\tau_{0}+K∙\gamma^{n}$$

where τ was shear stress (Pa), τ_0_ was yield stress (Pa), K was consistency coefficient (Pa^·sn^), γ was shear rate (S^−1^) and n was flow behaviour index, indicating the extent of deviation from Newtonian behaviour.

For viscoelasticity measurement, the strain amplitude was set as 1 %. Dynamic frequency sweep tests were measured from 0.1 Hz to 10 Hz within the linear viscoelastic range. Data obtained were storage modulus (*G'*) and loss modulus (*G"*).

The moduli of storage and loss were calculated as a power function of the oscillatory frequency (x), as is commonly used to describe the viscoelastic behaviour of food and dispersions (Rao et al., 1999).

(5) $$G^{\prime }=k^{\prime }∙\omega^{n^{\prime }}$$

(6) $$G^{\prime \prime }=k^{\prime \prime }∙\omega^{n^{\prime \prime }}$$

### 2.3 Cell wall polysaccharide isolation

#### 2.3.1 Alcohol insoluble solid preparation

Alcohol insoluble solids (AIS) powder were isolated using the same procedure as our previous study (Liu et al., 2023). Apple cubes, from the same batch used to make the purees, were frozen in liquid nitrogen and blended. Ethanol (70 %) was added and incubated for 1 h at room temperature. The mixture was then filtered through filter paper (15–20 μm). The insoluble material was mixed with 70 % ethanol, and the process was repeated three times. After that, the residue was rinsed with acetone, air-dried at 40 °C overnight, weighed, and stored in a desiccator for later fractionation.

#### 2.3.2 Sequential polysaccharide extraction

AIS (2 g) was suspended in 100 mL of distilled water and stirred for 1 h at 80 °C. The suspension was filtered using a Buchner funnel, and the insoluble material was washed twice. To obtain the water-soluble solids (WSS) fraction, the supernatant was collected, dialyzed against demi-water to remove free sugars with 3.5KDa dialysis membrane (Repligen Corporation, Boston, United States), and freeze dried.

The residue of the WSS extraction was mixed with 100 mL of chelating buffer (0.05 M EDTA and 0.05 M NH_4_ oxalate in 0.05 M NaOAc, pH = 5.2) and heated at 70 °C for 1 h to extract the chelating-agent soluble solid (ChSS). Filtered suspension and twice-washed residue were used. ChSS was produced by dialysing the supernatant for 18 h against an NH_4_-acetate buffer (pH 5.2), followed by another 18 h of dialysis against demi-water.

### 2.4 Analytical methods for pectin characterization

#### 2.4.1 Neutral sugar composition and galacturonic acid content

AIS, WSS, and ChSS samples were pre-treated for 1 h at 30 °C with 72 % (*w*/w) H_2_SO_4_ before being hydrolysed for 3 h at 100 °C after the acid was diluted to 1 M (Saeman et al., 1945). The neutral sugar content was then assessed using GC as alditol acetates and inositol as the internal standard (Englyst & Cummings, 1984). The GalA content was determined using the automated colorimetric *m*-hydroxydiphenyl assay, in which GalA reacts with *m*-hydroxydiphenyl to produce a colored complex that can be measured spectrophotometrically. GalA was used to create a calibration curve (Blumenkrantz & Asboe-Hansen, 1973; Thibault, 1979). All analyses were performed in duplicate.

#### 2.4.2 Degree of methyl-esterification

Each sample (5 mg) was saponified in 1 mL of 0.1 M NaOH for 1 h at 4 °C before being stored at room temperature overnight to assay DM. The amount of methanol emitted was determined using headspace GC (Huisman et al., 2004). Each analysis was performed twice, and blanks were incubated with water before analysis.

### 2.5 Enzymatic fingerprinting

#### 2.5.1 Pectin digestion

Endo-polygalacturonase (*endo*-PG) from *Kluyveromyces fragilis* (Daas et al., 1999) and pectin lyase (PL) from *Aspergillus niger* (Harmsen et al., 1990) were employed to hydrolyse the pectin fraction to perform enzymatic fingerprinting (Jermendi et al., 2022; Liu et al., 2023). Pectin fractions (5 mg) were diluted in 1 mL of 0.05 M NaOAc buffer (pH 5) and treated with enough PL and endo-PG to break down their corresponding substrates into oligomers in 6 h. All samples were incubated at 40 °C in a head-over-tail rotation for 24 h. The first 6 h were spent with PL, followed by 18 h with endo-PG. After inactivating the enzyme for 10 min at 100 °C, it was centrifuged (20,000 *g*, 15 min, 20 °C) to collect the supernatant for further investigation.

#### 2.5.2 High performance anion exchange chromatography

High performance anion exchange chromatography (HPAEC; Thermo Scientific, ICS5000) equipped with a CarboPac PA-1 column (ID 2 mm 250 mm) and a CarboPac PA guard column (ID 2 mm 25 mm) with pulsed amperometric detection (PAD; Dionex) and UV detection at 235 nm (Dionex) was used to quantify oligosaccharides digests. The temperature of the column was set to 20 °C, and samples (10 μL) were injected. The elution gradient was as described by Jermendi et al. (2022) and Liu et al. (2023), and the results were evaluated with Chromeleon 7.2 (Thermo Scientific)(Jermendi et al., 2022; Liu et al., 2023). Saturated GalA monomer, dimer, and trimer amounts were estimated using standards (Sigma-Aldrich, Steinheim, Germany). The response factors of the corresponding saturated GalA oligomers were used to calculate unsaturated GalA di- and trimers. The response from the tri-GalA standard was utilized to quantify all oligomers above DP3 (Jermendi et al., 2022; Liu et al., 2023).

#### 2.5.3 Hydrophilic interaction liquid chromatography

Hydrophilic interaction liquid chromatography (HILIC) was applied to identify and quantify specific oligomers, pectin digests were diluted with 50 % (*v*/v) acetonitrile and analyzed using a Vanquish UHPLC system (Thermo Scientific) outfitted with an Acquity UPLC BEH Amide column (1.7 m, 2.1 mm 150 mm) in conjunction with a Van Guard precolumn (1.7 m, 2.1 mm 5 mm). The temperature was 40 °C and the flow rate was 0.4 mL/min. The mobile phase gradient employed in this study was consistent with that used in our previous research (Liu et al., 2023). The scan range for acquiring mass spectra was set from *m*/*z* 300 to 2000 in negative mode. Thermo Scientific's Xcalibur 2.2 software was used to process the data.

#### 2.5.4 Descriptive parameters

Based on the quantity of non-methyl-esterified mono-, di-, and tri-GalA residues released by *endo*-PG, the DB and DB_abs_ were calculated. The DB and DB_abs_ are used to assess the proportion of non-methyl-esterified GalA monomers, dimers, and trimers liberated by endo-PG relative to the overall quantity of non-methyl-esterified GalA or the total GalA present in pectin, respectively. They were expressed using the following formulas (Jermendi et al., 2022; Liu et al., 2023):

(7) $$\mathrm{DB}=\frac{\sum_{n=1-3}{\left[\mathrm{Saturated}\;\mathrm{Gal}A_{n}\;\mathrm{Released}\right]}_{\mathrm{non}-\mathrm{esterified}}*n}{\mathrm{Total}\;\left[\mathrm{non}-\mathrm{esterified GalA in the polymer}\right]}*100$$

(8) $$\text{DBabs}=\frac{\sum_{n=1-3}{\left[\mathrm{Saturated}\;\mathrm{Gal}A_{n}\;\mathrm{Released}\right]}_{\mathrm{non}-\mathrm{esterified}}*n}{\mathrm{Total}\;\left[\mathrm{GalA in the polymer}\right]}*100$$

The quantities of GalA in saturated or unsaturated pectic methyl esterified oligosaccharides were used to determine DB_PGme_ and DB_PLme_ respectively. DB_PGme_ encompassed all partially saturated methyl-esterified GalA oligomers and DB_PLme_ quantified all esterified unsaturated GalA oligomers following PL degradation. The parameters were expressed using the formulae below (Jermendi et al., 2022; Liu et al., 2023):

(9) $$\text{DBPGme}=\frac{\sum_{n=3-8}{\left[\mathrm{Saturated}\;\mathrm{Gal}A_{n}\;\mathrm{Released}\right]}_{\mathrm{esterified}}*n}{\mathrm{Total}\;\left[\mathrm{GalA in the polymer}\right]}*100$$

(10) $$\text{DBPLme}=\frac{\sum_{n=2-8}{\left[\mathrm{Unsaturated}\;\mathrm{Gal}A_{n}\;\mathrm{Released}\right]}_{\mathrm{esterified}}*n}{\mathrm{Total}\;\left[\mathrm{GalA in the polymer}\right]}*100$$

### 2.6 Statistic

The “corrplot” tool included in R (Version 4.2.0) was used to perform correlation analysis (Pearson test). The significance threshold was established at *P* < 0.05. The “factoextra” package in R was used to perform K-means clustering and principal component analysis (PCA). In the statistical analysis, pectin structure indices—comprising sugar composition and content (mol%) and methyl-ester distribution parameters—were treated as independent variables. Meanwhile, physicochemical properties, including fruit dry matter, pulp content, total soluble solids, turbidity, stability, Zeta potential, particle size distribution, and rheology-related indices, were considered dependent variables representing puree quality indices.

## 3 Results and discussion

### 3.1 Apple puree characterization

**Fig. 1:**
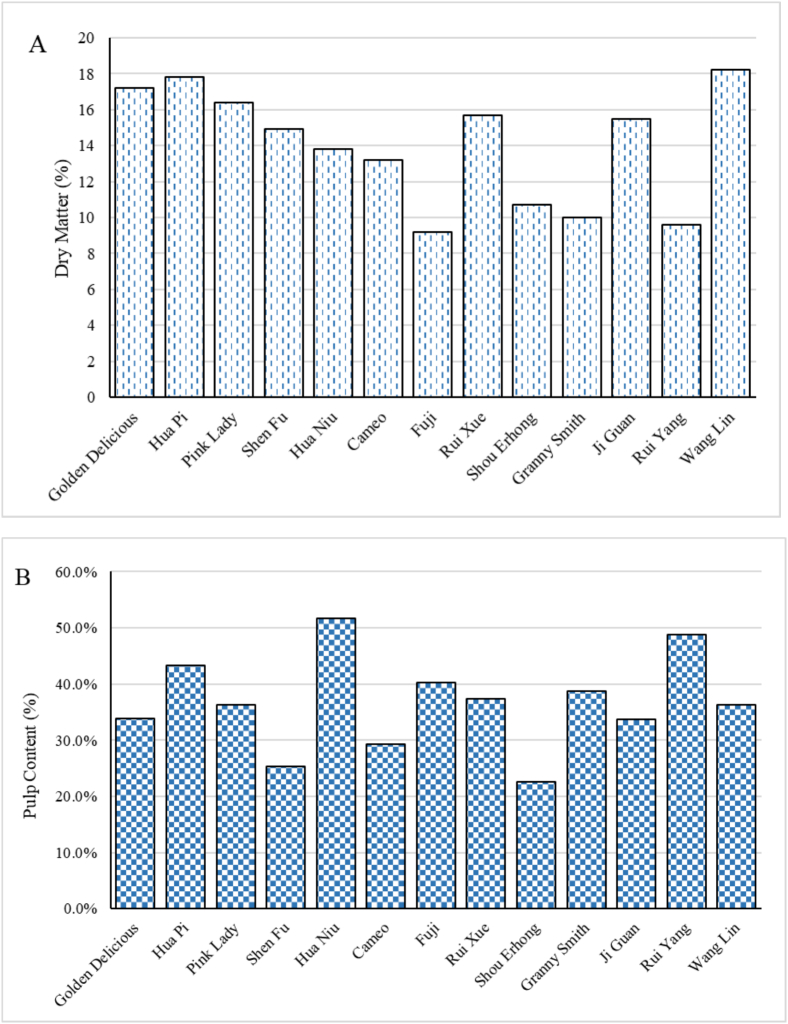

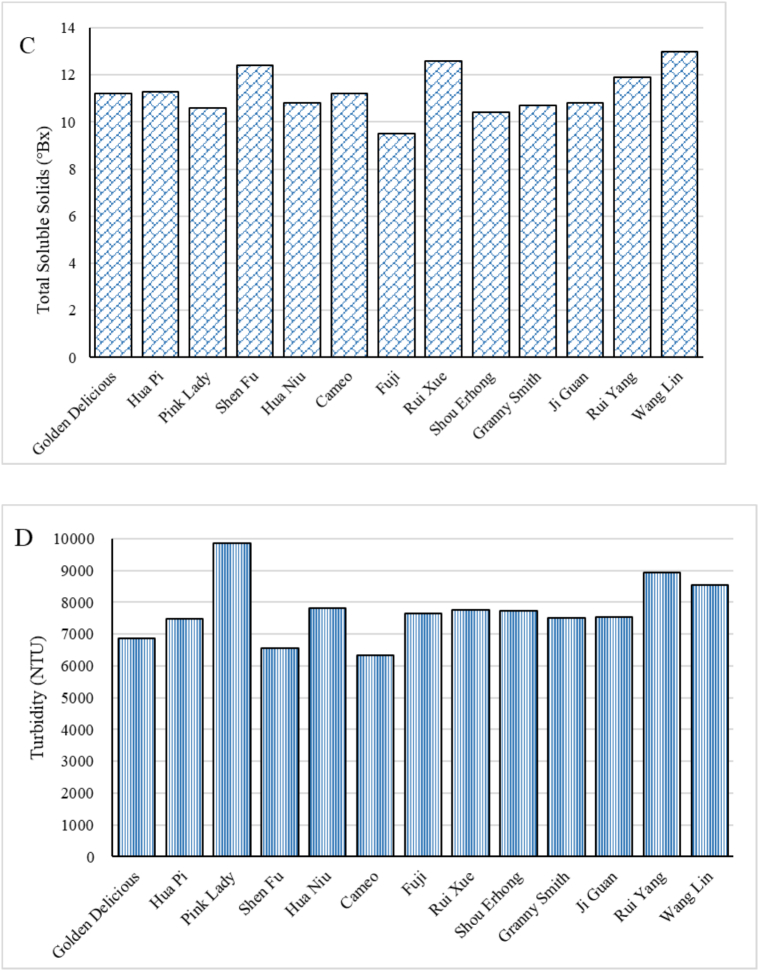

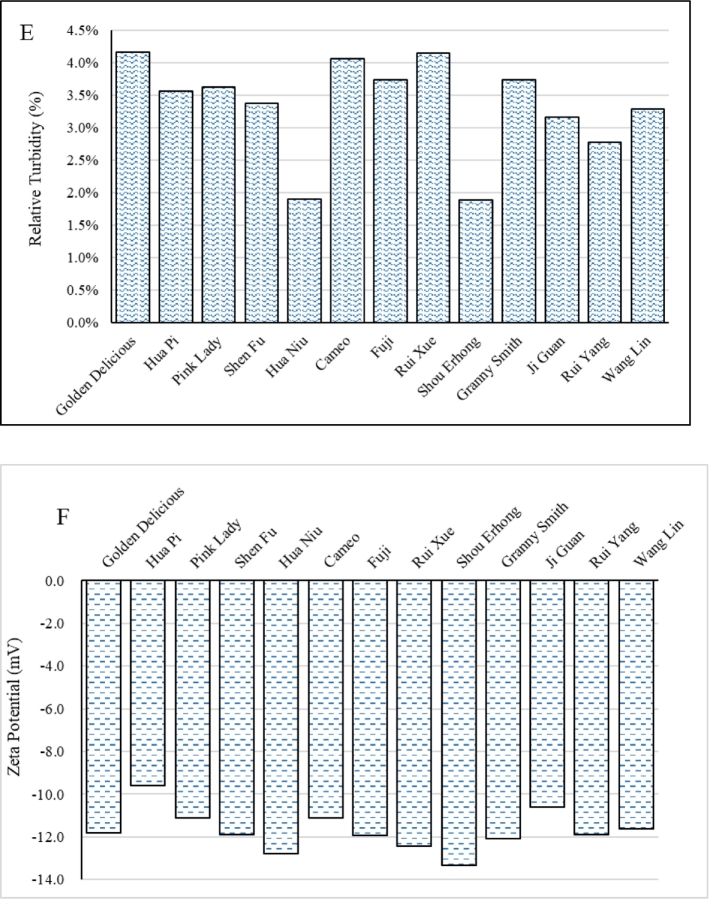
Physicochemical properties of different apple and apple purees, A:Dry matter of apple; B: Pulp content of apple puree; C: TSS of apple puree, D: Turbidity of apple puree; E: Stability of apple puree; F: ζ-potential of apple puree.

The physicochemical properties of apples are known to be linked to the sensory quality aspects of the puree. Values for all 13 apple cultivars of fruit dry matter, pulp content, total soluble solids, turbidity, stability, Zeta-potential, particle sizes distribution and rheology related indices are presented in Fig. 1 and Table 1.

**Table 1:** Particle sizes of apple purees from 13 apple cultivars and parameters of the Herschel-Bulkley and Power-Law Models Correlating Storage Modulus (G') and Loss Modulus (G").

Samples	Particle Size (μm)			Viscostiy			*G'*		*G"*	
	D[3,2]	D[4,3]	D50	τ_0_ (Pa)	k (Pa·s^n^)	n	k’ (Pa·s^n^)	n’	k” (Pa·s^n^)	n”
**Average**	**158.95**	**288.12**	**94.72**	**1.86**	**8.80**	**0.35**	**369.72**	**0.03**	**46.52**	**0.12**
Golden Delicious	173.85 ± 0.78	321.2 ± 1.27	83.4 ± 0.4	1.50	9.05	0.33	249.01	0.05	27.30	0.22
Hua Pi	184.25 ± 1.63	339.8 ± 5.37	130.65 ± 0.49	3.79	14.62	0.31	892.79	0.05	170.17	0.12
Pink Lady	151 ± 0.57	270.1 ± 0.71	74.28 ± 0.59	1.23	28.95	0.25	362.59	0.03	38.65	0.10
Shen Fu	149.6 ± 0.42	276 ± 2.12	69.76 ± 0.45	0.63	2.50	0.46	68.50	0.01	7.56	0.18
Hua Niu	203.35 ± 0.21	387.65 ± 1.63	118.6 ± 0.42	4.35	8.30	0.30	415.47	0.03	62.30	0.08
Cameo	152.7 ± 0.28	276.5 ± 0.14	73.4 ± 0.01	2.35	5.23	0.35	467.48	0.03	45.92	0.11
Fuji	169.95 ± 0.21	313.65 ± 0.49	84.97 ± 0.21	2.26	9.87	0.34	565.56	0.05	62.73	0.06
Rui Xue	137.35 ± 0.07	244.6 ± 1.13	73.1 ± 0.83	1.19	7.74	0.32	340.80	0.03	37.63	0.06
Shou Erhong	156.3 ± 1.41	255.9 ± 1.27	162 ± 2.26	0.65	1.83	0.41	51.96	0.03	5.43	0.17
Granny Smith	132.5 ± 0.28	235.2 ± 3.54	72.62 ± 2.23	0.02	5.18	0.33	458.94	0.05	50.42	0.11
Ji Guan	141.1 ± 1.27	266.25 ± 0.35	68.53 ± 0.76	1.83	6.96	0.39	283.72	0.03	34.90	0.14
Rui Yang	154.15 ± 1.2	281.05 ± 2.05	71.99 ± 1.65	0.64	5.44	0.42	274.38	0.03	26.69	0.10
Wang Lin	160.2 ± 0.57	277.65 ± 1.48	148.05 ± 2.76	3.78	8.72	0.32	375.19	0.03	35.09	0.14

τ_0_: Yield stress.

k: Consistency index.

n, n’,n”: Flow behaviour index.

#### 3.1.1 Dry matter content of the apples and pulp content of apple purees

The dry matter contents of each apple cultivar ranged from 9.2 to 18.2 % as shown (Fig. 1A). The dry matter content for each variety indicates significant variability that can influence apple processing suitability and sensory properties of apple products.

The pulp content is another factor that could affect the sensory properties of apple purees and may even lead to distinct applications. As shown in Fig. 1B, the pulp content of the 13 apple purees ranged from 22.7 to 48.7 %, mostly in the range of 30 % – 40 % (36.7 % on average). The variation in pulp content among different fruit pulps may be attributed to differences within apple variety, which encompass variations in water content, polysaccharide level, and the structure of cell wall materials. The pulp content range was higher than the previous work by Espinosa-Muñoz et al. which reported a value of 28.7 % (Espinosa-Muñoz et al., 2013). Generally, purees with a higher pulp content would be thicker and more viscous (Espinosa-Muñoz et al., 2013). As the pulp content values were measured by the centrifugation of apple puree, the mass of sediment (pulp weight) will certainly be influenced by the bound amount of water. During the processing, the pectin in apple cells might be solubilized.

#### 3.1.2 Total soluble solids of apple puree

The total soluble solids (TSS) values of apple puree ranged from 9.5 to 13°Brix, averaging 11.3°Brix (Fig. 1C), indicating a consistent level of sweetness across cultivars. The Wang Lin puree had the highest TSS, suggesting a greater concentration of sugars and enhancing its flavour profile, while the Fuji variety had the lowest TSS, indicating a milder sweetness. These differences in TSS highlight the influence of apple variety on puree characteristics, affecting consumer preferences.

#### 3.1.3 Turbidity and relative stability of apple puree

The high turbidity levels (Fig. 1D) observed indicate the presence of a significant amount of pulp, fibres, or other solids in the purees. The turbidity of the purees ranged from 6320 to 9850 NTU, indicating differences in the concentration of suspended particles or solids within the purees. The *T*_*rel*_ values are used to highlight the stability of apple purees (Fig. 1E). A puree with higher *T*_*rel*_ values indicates that it was more difficult to separate the puree's liquid and pulp, representing a more stable puree. For instance, Shou Erhong puree had the lowest *T*_*rel*_ value (1.89 %) showing that the liquid/pulp separation was much easier than for Golden Delicious puree with a *T*_*rel*_ value of 4.16 %. There appears to be no clear relationship between turbidity and stability. For example, Shou Erhong exhibited a high turbidity but the lowest stability, while Pink Lady had the highest turbidity and yet a normal stability value.

#### 3.1.4 Zeta-potential and particle size distribution of apple puree

In liquid food systems like purees, macromolecules will interact and influence texture (Chevalier et al., 2019). The Zeta-potential was measured to assess colloidal stability. A negative Zeta-potential, ranging from −9.6 to −13.3 mV (Fig. 1F), helps prevent particle aggregation and maintains a homogeneous texture. This electrostatic repulsion supports stability by resisting phase separation and sedimentation, crucial for consistent food products.

To evaluate particle size distribution in apple purees, we measured the area-based diameter (D[3,2]), volume-based diameter (D[4,3]), and median particle diameter (D50) (Table 1). As can be seen from the particle size distribution curves (Fig. S1), all purees exhibited bimodal or even multimodal distributions, which indicate complexity sizes of particles. Although the particle size distributions were not identical, a generality can still be found. It can be seen that all the puree had abundant distributions in 248, 88 and 22 μm. The results suggest that although the particle size distributions of the puree vary in percentage, the influence of processing methods on particle size is generally consistent. Therefore, the variations in distribution may be attributed to differences in the properties of the materials forming the particles, such as pectin. Particle size significantly affects the rheological properties of purees and relates to the cell wall's water-binding capabilities (Buergy et al., 2020; Leverrier et al., 2016).

D[3,2] values ranged from 132.5 to 203.35 μm (average: 158.9 μm), and D[4,3] values ranged from 235.2 to 387.65 μm (average: 288.1 μm). A lower area-based diameter typically correlates with a lower volume diameter, potentially impacting taste. D50 values ranged from 68.53 to 148.05 μm (average: 94.7 μm). Some purees, like the Pink Lady and Shou Erhong, showed similar D[4,3] and D[3,2] values but differed in D50, indicating different particles size in various apple puree which leading to variations in mouth feel of the product (Espinosa et al., 2011). Additionally, smaller particle sizes contribute to lower Zeta-potential and greater stability (Zhu et al., 2020).

#### 3.1.5 Rheological analysis

The rheological properties are crucial in determining the ability of puree processing and its overall quality when consumed. Due to the presence of polysaccharides, apple cultivars display variations in viscosity and viscoelastic indexes, which directly impact the properties of their purees. The Herschel-Bulkley model was used to express the rheological parameters. The yield stress and flow behaviour index differed among the apple purees, whereas the consistency index showed a relatively larger variation (Table 1). The yield stress ranged from 0.02 to 4.35 Pa (1.86 Pa on average), indicating significant variability in their flow behaviour and texture properties. These relatively low yield stress values indicated weak internal structures that can be broke down easily, allowing the puree to deform and with a liquid-like flow behaviour. The yield stress of the puree is reported to be affected by the pulp content, and even a small change in pulp content can strongly affect the values (Espinosa et al., 2011). All apple purees have flow behaviour indexes under 1, indicating the purees were shear thinning and all behaved as non-Newtonian fluids. The consistency index (k) ranged broadly from 1.83 to 28.95 (average 8.80), indicating that the viscosity of the apple purees significantly differed within cultivars. Such differences will significantly affect the texture, mouthfeel, and overall quality of the purees. For instance, the k value of the Pink Lady (28.95) puree indicated that the puree was thicker and much more viscous than the Shou Erhong (1.83) puree. Purees with a different viscosity behaviour could be used in different food applications. Puree with a higher consistency index might be used for fillings, while a lower consistency index puree might be more suitable for elderly and infant consumers. The purees exhibited yield stress and displayed a weaker gel-like behaviour in terms of their higher elastic moduli (G′) compared to their loss moduli (G″). The apple purees showed different mechanical responses to deformations due to the huge diversity in the two moduli (Table 1). This could be due to variations in the composition or structure of the endogenous pectin in apple purees, which widely exists in apple puree (Varela et al., 2007). In summary, pectin as present in apples has gel-forming and thickening properties, which can affect the viscosity characteristics of the apple puree.

### 3.2 Pectic polysaccharide structure characterization

The cell wall material was extracted in a mild way by ethanol washing out of sugars and other small molecules. The cell wall material was further fractionated to obtain fractions for water soluble pectin and for chelating agent soluble pectins. Structure differences were found in these pectin fractions from different apple cultivars as the results of the yield of each polysaccharide fraction, sugar composition and content, and degree and distribution of methyl-esters were shown.

#### 3.2.1 Yield, content and sugars composition of each polysaccharide fraction

The yields of each polysaccharide fraction are presented in Table 2. The AIS content varied among different apple cultivars, ranging from 1.8 to 3.8 g (average 2.3 g) per 100 g of fresh mass. The yield of the WSS fraction ranged from 124.8 to 355.6 mg (average 211 mg) per 100 g of fresh mass, while the ChSS fraction yielded between 342 and 1158 mg (average 694 mg) per 100 g of fresh mass. Wang Lin cultivar exhibited the highest yield in AIS, as well as the in WSS and ChSS. In contrast, the Hua Niu cultivar, which had the lowest AIS content, showed average levels of WSS and even exceeded the average value for ChSS level. This yield range is similar to our previous research and by Li et al., which reported comparable results for five different apple cultivars (Li et al., 2020). It is evident that different apple cultivars exhibit a wide range of yields in AIS, WSS, and ChSS fractions and no correlation was found between AIS content and the yields of WSS and ChSS.

**Table 2:** Yields of cell wall polysaccharides and the pectin fractions derived from 13 apple cultivars.

Samples	AIS	WSS	ChSS
	g/100 g fresh fruit	mg/100 g fresh fruit	mg/100 g fresh fruit
**Average**	**2.3**	**211**	**694**
Golden Delicious	2.1	231	690
Hua Pi	3	356	741
Pink Lady	2.6	208	880
Shen Fu	1.8	166	764
Hua Niu	1.8	217	767
Cameo	1.9	231	573
Fuji	1.8	125	368
Rui Xue	2.4	133	664
Shou Erhong	2.1	133	684
Granny Smith	2.7	231	925
Ji Guan	2.3	209	342
Rui Yang	1.9	170	459
Wang Lin	3.8	332	1158

AIS: Alcohol insoluble solids, WSS: Water-soluble solids, ChSS: Chelating-soluble solids.

The sugar content and composition of all fractions for the individual varieties is shown in Table 3. For all AIS fractions, galacturonic acid, pectic polysaccharides and glucose were the dominant sugars. Galacturonic acid (GalA) contents ranged from 30 to 42 mol%, with 35 mol% as average. The amount of glucose ranged from 28 to 46 mol%, with an average of 35 mol%. Xylose had a rather similar level in AIS from each apple cultivar, ranging from 6 to 10 mol%. Arabinose amounts in each cultivar ranged from 7 to 16 mol%. The higher content of GalA, glucose and xylose indicated that apple cell walls were mainly composed of pectin, cellulose and xyloglucan, as described by a previous study (Renard et al., 1991). Rhamnose, fucose, mannose and galactose were present in lower relative amounts. The high ratio of GalA indicated the pectic structure mainly consists of homogalacturonan elements.

**Table 3:** Sugar composition (mol%) and degree of methyl-esterification of AIS, WSS and ChSS fractions from 13 apple cultivars.

Samples	Rha	Fuc	Ara	Xyl	Man	Gal	Glc	Uronic acid	DM
									(%)
AIS									
Golden Delicious	1.5	1.9	10.8	8.7	2.4	5.8	37.3	31.6	57
Hua Pi	1.6	1.9	10.8	8.4	2.9	4.2	32.0	38.3	58
Pink Lady	1.6	1.8	12.0	8.9	3.0	5.1	29.3	38.3	57
Shen Fu	1.3	1.6	8.5	9.0	2.2	5.4	36.5	35.5	61
Hua Niu	1.5	1.2	6.7	9.1	2.7	3.1	33.7	42.0	63
Cameo	1.3	1.6	6.5	7.6	1.6	4.8	45.6	30.9	59
Fuji	1.3	1.4	10.2	7.3	2.3	5.2	38.0	34.4	66
Rui Xue	1.5	1.7	13.9	9.4	2.3	5.0	31.8	34.5	64
Shou Erhong	1.3	1.4	7.8	9.7	1.9	4.7	39.4	33.8	66
Granny Smith	1.0	1.5	16.3	6.1	2.6	7.5	30.5	34.6	62
Ji Guan	1.3	1.6	10.1	7.8	2.5	4.7	37.3	34.8	63
Rui Yang	1.3	1.6	14.9	8.9	2.0	5.3	28.1	37.8	64
Wang Lin	1.5	1.6	10.9	7.7	2.6	7.7	38.4	29.7	62
WSS									
Golden Delicious	1.9	0.6	9.0	1.7	1.5	7.2	8.5	69.7	77
Hua Pi	1.2	0.0	6.3	0.8	1.0	4.8	7.8	78.3	75
Pink Lady	2.0	0.0	7.5	1.7	1.9	6.6	13.8	66.4	75
Shen Fu	2.2	0.0	8.9	2.3	1.9	9.1	9.9	65.7	78
Hua Niu	1.5	0.0	5.6	0.6	0.5	5.0	5.5	81.3	79
Cameo	1.1	0.0	5.5	0.2	0.0	5.7	5.2	82.3	79
Fuji	1.4	0.0	5.5	0.0	1.2	9.9	2.7	79.3	76
Rui Xue	1.5	0.0	5.3	0.0	0.0	7.2	1.3	84.7	64
Shou Erhong	1.8	0.0	5.6	0.8	0.0	6.8	1.7	83.3	70
Granny Smith	1.5	0.0	17.5	0.7	2.3	9.1	9.7	59.2	68
Ji Guan	1.8	0.0	8.3	1.5	1.4	5.3	6.5	75.2	69
Rui Yang	2.2	0.0	13.4	3.5	1.4	13.2	9.9	56.4	71
Wang Lin	2.3	0.0	6.2	1.9	0.4	7.0	3.9	78.4	69
ChSS									
Golden Delicious	2.7	0.0	12.4	2.4	0.0	3.4	0.0	79.1	37
Hua Pi	2.5	0.0	9.9	1.7	0.0	2.6	0.0	83.4	54
Pink Lady	1.3	0.0	6.1	1.0	0.0	3.4	0.0	88.1	38
Shen Fu	0.9	0.0	5.7	0.0	0.0	1.7	0.0	91.7	29
Hua Niu	1.0	0.0	5.3	0.0	0.0	1.3	1.1	91.3	31
Cameo	1.2	0.0	7.9	1.1	0.0	3.2	0.0	86.6	44
Fuji	1.0	0.0	5.6	0.3	0.0	1.5	0.0	91.4	71
Rui Xue	1.3	0.0	9.0	1.6	0.0	2.2	2.3	83.5	75
Shou Erhong	1.4	0.0	6.0	1.1	0.0	2.2	0.0	89.3	78
Granny Smith	0.7	0.0	10.6	0.0	0.0	3.3	0.0	85.3	60
Ji Guan	1.0	0.0	8.2	1.0	0.0	2.0	1.1	86.6	31
Rui Yang	0.9	0.0	5.2	0.0	0.0	3.9	0.0	90.0	52
Wang Lin	2.1	0.0	12.2	1.8	0.0	1.6	1.5	80.9	68

AIS: Alcohol insoluble solids, WSS: Water-soluble solids, ChSS: Chelating-soluble solids.

Rha: rhamnose, Fuc: fucose, Ara: arabinose, Man: mannose, Gal: galactose, Glc: glucose, GalA: galacturonic acid, DM: Degree of methyl-esterification.

For both WSS and ChSS fractions, the major sugar in the polysaccharides is GalA, both can be considered as pectin fraction. In WSS fractions, the GalA accounts for 57–85 mol% of the total sugar amounts. The relatively low proportion of rhamnose (1–2 mol%) suggests that the pectins have relatively lower levels of the RG-I structural element, in slightly different amounts. The side chain sugars arabinose and galactose varied in WSS fractions from 5 to 18 mol% and 5–13 mol%, respectively. The high GalA contents indicated the linear structure of the WSS fractions with some arabinan and galacturonan side chains. In purees, pectin with a more linear structure is more likely to form a weaker gel and has lower viscosity enhancing properties compared to highly branched pectin (Wu et al., 2024). This means that the linear structure of WSS pectin may contribute to lower thickness and smoothness of the puree texture. For instance, the apple Rui Yang puree showed a low GalA content of 56 % and a relatively high Rha content of 2 % in the WSS fraction, which might contribute to lower viscosity, as detailed in Table 1. In contrast, the apple Cameo puree exhibited higher viscosity, with a greater GalA content of 82 % and 1 % Rha content. This suggests that even within “linear” pectin, factors such as chain length, methylation, and interactions with other polymers in the puree could unexpectedly affect viscosity.

The ChSS fractions had a high amount of GalA, all fractions had over 80 mol% of GalA. The average rhamnose content in the ChSS fractions was lower than in the WSS, indicating that the RG-I backbones in ChSS fractions are shorter than those in WSS. For the side chain sugars, the arabinose levels (5–12 mol%) was similar to the levels found in WSS, whereas galactose levels (1–4 mol%) were lower. By comparing different sugar ratios (Table S1), an overview of pectin spatial structures can be obtained. As indicated by the RG-I percentage, the WSS fractions (13.2% – 31 %, with an average of 18.9 %) generally exhibited a more branched structure than the ChSS fraction (8.6 % - 21.2 %, with an average of 13.3 %). Furthermore, the ChSS fractions tended to have a more linear structure than the WSS fractions, as indicated by the HG to RG-I ratio (36.5 on average versus 22.4 on average). The average side chain lengths showed that the side chain lengths were quite similar in both the WSS and ChSS fractions, with average values of 9.2 and 8.5, respectively. Zooming in on the sugar ratios of pectin fractions from each apple cultivar reveals differences in the side chain proportions and lengths. These variations may lead to differing physical properties of the pectin. In comparison to our previous study with a partly different series of apple varieties, the sugar content in the AIS, WSS, and ChSS fractions was mostly within a similar range (Liu et al., 2023). The only exception was the xylose content in AIS, which was relatively lower than in the earlier study. This suggests a difference in the xyloglucan content of the AIS polymers between the two batches.

#### 3.2.2 Degree of methyl-esterification of pectic polysaccharides

In addition to sugar composition, methyl-esterification is an important variation in native apple pectin populations. The DM values of all fractions are also shown in Table 3. For AIS fractions, the DM values are in a relatively narrow range of 57 % to 66 % (average: 61 %), which means that all AIS pectins can be classified as high-methyl-esterified pectins. The average DM values were slightly lower than those found in earlier studies on Golden Delicious apple with AIS DM of 75 % (Billy et al., 2008). The average DM of WSS (64–79 %) was much higher than that of AIS, and WSS pectins can be regarded as high DM pectins. The DM values of WSS were slightly higher than found in our previous study with DM values mostly in a range of 51–70 % (Liu et al., 2023). For ChSS pectins, the DM values (29 % – 78 %) varied among the different apple cultivars. It is essential to note that all ChSS pectins were bound to calcium in the cell wall, and they could only be extracted using a chelating buffer by disrupting the calcium-pectin complex. As calcium-bound pectins, ChSS fractions typically exhibit a relatively low degree of methyl-esterification (DM) to meet the requirement of having 6–13 continuous unsubstituted GalA residues within the HG backbone in order to initiate calcium-pectate cross-linking (Luzio & Cameron, 2008). The significant disparity of the DM for ChSS pectins allows them to categorize them into two distinct populations: one as high-DM pectin (>50 %) and the other as low-DM pectin (<50 %). An example for the high-DM pectate gelation is the ChSS fractions from e.g. the variety Shou Erhong with a relatively high DM (76 %) and still being calcium sensitive. Obviwusly, despite the high DM, the gelation indicates clearly the presence of continuous unsubstituted GalA regions within its HG backbone. As mentioned, the DM of the pectin in the apple puree is known to have an effect on the texture of the final product (Buergy, Rolland-Sabaté, Leca, Falourd, et al., 2021). However, the disparity between the DM found for the two groups of ChSS pectins may result in rather similar texture characteristics for the ChSS pectins in the final puree and violate the general rule by Buergy et al., 2021a that DM directly relates with the structure (Buergy, Rolland-Sabaté, Leca, Falourd, et al., 2021).

#### 3.2.3 Methyl-ester distribution pattern of extracted pectins

The methyl-ester distribution in pectins is crucial for their functionalities (Guillotin et al., 2005; Jermendi et al., 2022). Using enzymatic fingerprinting, the methyl-ester distribution can be quantified and compared (Liu et al., 2023). Oligomers released post-enzyme treatment are identified and quantified via High Performance Anion-Exchange Chromatography and Hydrophilic Interaction Liquid Chromatography with mass spectrometry (HPAEC & HILIC-MS). Endo-polygalacturonase (*endo*-PG) from *A. aculeatus* can hydrolyse the α-1,4 glycosidic bonds between non-methyl-esterified GalA units in HG, requiring at least 4 unesterified GalA sequences in line and producing a mixture of methyl-esterified and non-methyl-esterified GalA oligomers. The end products of a long block of unesterified GalA residues would be unesterified mono-, di-, and triGalA. Pectate lyase (PL) from *A. niger* targets the pectin structure, cleaving between methyl-esterified GalA residues in the HG region through a β-elimination reaction and release full and partly methylesterified unsaturated GalA oligomers DP ≥2. Quantification of the various oligomers facilitates the calculation of parameters like DB, DB_abs_, DB_PGme_, and DB_PLme_, offering a detailed assessment of methyl-ester distribution. The DB and DB_abs_ assess the proportion of non-methyl-esterified GalA monomers, dimers, and trimers released by endo-PG relative to the total non-methyl-esterified or total GalA in pectin. DB_PGme_ includes all partially saturated methyl-esterified GalA oligomers, while DB_PLme_ quantifies all esterified unsaturated GalA oligomers after PL degradation.

Table 4 shows WSS pectins' distribution parameters, with DB_abs_ values mostly between 2 % and 4 %, except for Rui Yang (15 %). Highly methyl-esterified WSS pectins had low DB and DB_abs_ values, indicating significant unsubstituted GalA fractions. DB_PGme_ values ranged from 14 % to 36 %, reflecting esterified oligomers. Consequently, low DB and DB_abs_ resulted in higher DB_PLme_ values (52 % – 82 %). Rui Yang's high DB_PGme_ (36 %) aligns with its elevated DB and DB_abs_ values. Despite similar DM values among WSS fractions, DB_PGme_ and DB_PLme_ showed quite some variation, indicating differences in methyl-ester distribution. For instance, Ji Guan and Wang Lin had the same DM (69 %) but differed in DB_PLme_ (52 % vs. 75 %), indicating more dense methyl-esterified regions for the latter.

**Table 4:** Structural descriptive parametersof WSS and ChSS pectin fractions from 13 apple cultivars.

Samples	DM	DB (%)	DB_abs_ (%)	DB_PGme_ (%)	DB_Pgme_ (%)	DB_PGme_/DB_abs_	DB_PGme_/DB_PLme_	(DB_PGme_ + DB_abs_)/DB_PLme_
WSS								
Golden Delicious	77	14	3	20	82	6.0	0.2	0.3
Hua Pi	75	13	3	19	65	5.8	0.3	0.3
Pink Lady	75	13	3	22	67	7.0	0.3	0.4
Shen Fu	78	13	3	21	59	6.9	0.4	0.4
Hua Niu	79	9	2	20	79	10.8	0.2	0.3
Cameo	79	15	3	16	73	5.0	0.2	0.3
Fuji	76	10	2	14	78	5.5	0.2	0.2
Rui Xue	64	8	3	18	82	6.2	0.2	0.3
Shou Erhong	70	13	4	20	79	5.1	0.3	0.3
Granny Smith	68	10	3	25	70	7.4	0.4	0.4
Ji Guan	69	11	3	18	52	5.6	0.3	0.4
Rui Yang	71	50	15	36	79	2.5	0.5	0.6
Wang Lin	69	9	3	15	75	5.2	0.2	0.2
ChSS								
Golden Delicious	37	22	14	95	7	7.0	13.6	15.5
Hua Pi	54	20	9	70	25	7.7	2.8	3.2
Pink Lady	38	14	9	48	9	5.6	5.5	6.4
Shen Fu	29	17	12	81	5	6.8	17.8	20.4
Hua Niu	31	21	14	79	6	5.5	14.2	16.8
Cameo	44	20	11	83	13	7.4	6.2	7.0
Fuji	71	15	4	26	95	6.0	0.3	0.3
Rui Xue	75	22	5	32	82	5.8	0.4	0.5
Shou Erhong	78	25	5	12	78	2.2	0.2	0.2
Granny Smith	60	23	9	66	21	7.1	3.1	3.5
Ji Guan	31	16	11	65	19	5.9	3.4	4.0
Rui Yang	52	29	14	93	17	6.8	5.4	6.2
Wang Lin	68	12	4	29	85	7.2	0.3	0.4

WSS: Water-soluble solids.

ChSS: Chelating-soluble solids.

DM: Degree of methyl-esterification, mol of methanol per 100 mol of the total GalA.

DB: Degree of blockiness, the amount of non-esterified mono-, di- and tri- GalA per 100 mol of the non-esterified GalA in the sample.

DB_abs_: Absolute degree of blockiness, the amount of mono-, di- and tri- GalA per 100 mol of the total amount of GalA in the sample.

DB_PGme:_ Degree of blockiness by Endo-PG, the amount of saturated methyl-esterified oligomers released by *endo*-PG per 100 mol of the total GalA.

DB_PLme_: Degree of blockiness by PL, the amount of galacturonic acid residues in unsaturated oligomers released by PL per 100 mol of the GalA.

To further investigate the methyl-ester distribution, additional analysis of parameters was performed (Table 4). The DB_PGme_/DB_abs_ ratio (ranging from 2.5 to 10.8) indicates more partly methyl-esterified than non-methyl-esterified GalA regions, varying by variety. The DB_PGme_/DB_PLme_ ratio (range 0.2–0.5) highlights the prevalence of highly methyl-esterified segments. This supports the (DB_PGme_ + DB_abs_)/DB_PLme_ ratio (0.1–0.6), reflecting the relationship between endo-PG and PL degradable segments. Overall, these ratios illustrate the presence of some specific methyl-esterified GalA sequences as shown for the Rui Yang variety having a high DB_abs_, next to both a relatively high DB_PGme_ *and* DB_PLme_ and relatively more non-methyl-esterified blocks alongside highly methyl-esterified segments.

The separation of ChSS extracts from various apple varieties into low and high DM pectins resulted in significant variations in descriptive parameters. DB and DB_abs_ values ranged from 12 to 29 % and 4–14 %, respectively, both notably higher than in WSS fractions. High DM ChSS pectins (> 65 %) showed slightly higher DB_abs_ values than similar DM WSS fractions, indicating more non- or low-methyl-esterified blocks.

A broad range of DB_PGme_ (12–95 %) and DB_PLme_ (5–95 %) values was observed. High DM ChSS pectins had lower DB_PGme_ values (12–32 %), while low DM pectins had much higher values (48–95 %), leading to generally higher DB_PLme_ values for high DM pectins, except for Granny Smith, which had a high DM but low DB_PLme_.

The DB_PGme_/DB_abs_ ratio (2.2–7.7) indicated a predominance of partly methyl-esterified GalA segments. The ratios DB_PGme_/DB_PLme_ and (DB_PGme_ + DB_abs_)/DB_PLme_ varied widely, showing that high DM ChSS pectins contained densely packed non-methyl-esterified blocks, aligning with their interaction with calcium ions.

In apple puree from different varieties, the distinct methyl-ester distribution in water-soluble and calcium-bound pectin influences particle size distribution, contributing to a uniform texture in the final product.

### 3.3 Pectin structural characteristics clustering and correlations with puree properties

**Fig. 2:**
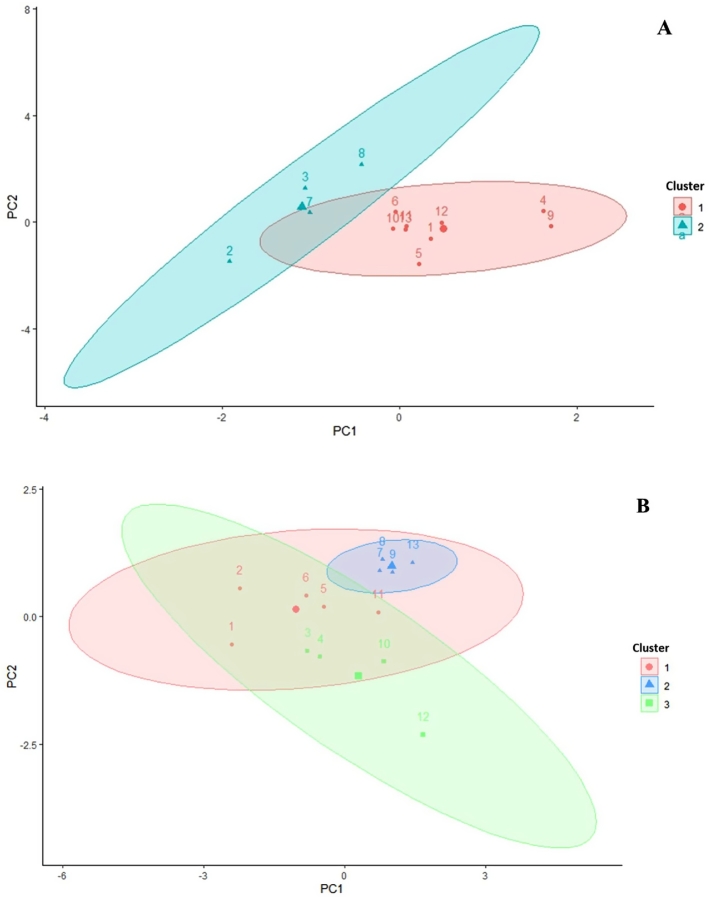
Principal component analysis (PCA) utilizing puree quality indices and pectin structural characteristics of 13 Chinese apple varieties examined the relationship between pectic polysaccharides and apple puree properties, clearly separating cultivars based on their pectin composition and textural outcomes, thus underscoring the predictive value of pectin structure. A: PCA clustering plot based on puree quality indices; B: PCA clustering plot based on pectin structural characteristics. Apple serial number: 1. Golden Delicious; 2. Hua Pi; 3. Pink Lady; 4. Shen Fu; 5. Hua Niu; 6. Cameo; 7. Fuji; 8. Rui Xue; 9. Shou Erhong; 10. Granny Smith; 11. Ji Guan; 12. Rui Yang; 13. Wang Lin. (For interpretation of the references to colour in this figure legend, the reader is referred to the web version of this article.)

Principal component analysis (PCA) was conducted using either puree quality indices or pectin structural characteristics. The analyses distinctly categorized cultivars based on their pectin composition and textural results, highlighting the predictive significance of pectin structure. PCA was not performed with all variables to avoid confounding effects and to ensure clearer insights by focusing on specific groupings of pectin structural characteristics and puree quality indices. The resulting clustering plots are illustrated in Fig. 2. PCA based on puree indices grouped the apple cultivars into two clusters (Fig. 2A): Cluster 1 included Golden Delicious, Shen Fu, Hua Niu, Cameo, Shou Erhong, Granny Smith, Ji Guan, Rui Yang, and Wang Lin, while Cluster 2 consisted of Hua Pi, Fuji, Pink Lady, and Rui Xue. These clusters represent groups of apple cultivars that share similar physicochemical properties.

In contrast, PCA using pectin structural characteristics revealed three distinct groups: Group 1 included Golden Delicious, Hua Pi, Hua Niu, and Ji Guan; Group 2 comprised Fuji, Rui Xue, Shou Erhong, and Wang Lin; and Group 3 included Pink Lady, Shen Fu, Granny Smith, and Rui Yang, as shown in Fig. 2B. The differences in grouping suggest that while some cultivars may share similar physical properties, they may possess different native pectin structures. These findings indicate that despite sharing similar physicochemical properties, the apple cultivars exhibit distinct native pectin structures, suggesting that the biochemical pathways responsible for pectin composition and modification can vary independently of the traits that influence puree quality, thus highlighting the complexity of fruit structure-function relationships.

Cluster 2 cultivars in the PCA analysis of apple puree quality (Hua Pi, Fuji, Pink Lady, Rui Xue) exhibited shared pectin features like a higher DM and a higher DB_PLme_ in ChSS fraction when compared to the apples in Cluster 1 (a mean DM of 59.5 % and 47.8 % and a mean DB_PLme_ of 52.8 % and 27.9 % for cluster 2 and 1 respectively). Notably, Fuji exhibited the highest DB_PLme_ value (95 %) among Group 2 cultivars in the PCA analysis using puree quality index, in contrast to Hua Pi's lower value (25 %) in the same cluster. The different methylester distribution may correlate with their distinct textural profiles: Hua Pi's puree displayed a larger particle size (D50 = 162.00 μm) compared to Fuji's (130.65 μm), likely due to fragmented pectin networks arising from uneven methyl-ester distribution. Meanwhile, Pink Lady's intermediate ChSS DM (38 %) and higher WSS rhamnose (2 %), lower arabinose and (8 %) and galactose (7 %) contents compared to the averages in Cluster 1 (1.8 %, 8.9 %, and 7.6 %, respectively), aligned with its transitional clustering behaviour, balancing gel rigidity (low DM pectin) and water retention (neutral sugar side chains).

**Fig. 3:**
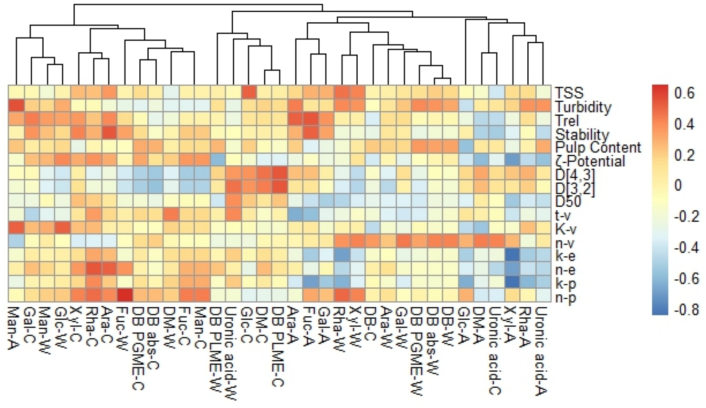
Cluster analysis of pectin characteristics and correlation heatmap between apple puree quality indices and apple pectin structure characterisations. -A: AIS; -W: WSS; -C: ChSS (e.g. DM-W = Degree of Methyl esterification of WSS); t-v: yield stress of Herschel-Bulkley model; K-v: consistency coefficient of Herschel-Bulkley model; n-v: flow behaviour index of Herschel-Bulkley model; k-e: consistency index of storage moduli; n-e: flow behaviour index of storage moduli; k-p: consistency index of loss moduli; n-p: flow behaviour index of loss moduli.

To gain an overall understanding of the correlations, a correlation analysis was performed to examine how pectin characteristics affect puree properties. As illustrated in the correlation heatmap (Fig. 3), the particle size of the puree shows a positive correlation with the methyl-ester distribution parameters of ChSS pectin, specifically the DM and DB_PLme_. This observation aligns with the fact that both the methyl-ester distribution parameters of ChSS and the puree's particle size significantly contribute to the same underlying variability in the PCA dataset.

As discussed in the previous section on pectin characterization (Table 4), the DB_PLme_ can differ between high and low DM ChSS fractions. This indicates that high DM ChSS pectins, particularly those containing significant amounts of highly methyl esterified GalA blocks, next to the non-methyl-esterified blocks causing interaction with calcium, may contribute to an increase in the particle size of the apple puree. This increase may occur because high DM pectin can aggregate in the puree, forming a second network through hydrophobic interactions and hydrogen bonding within a calcium gel.In the case of a low number of high methyl blocks, only the calcium pectate gel forming mechanism provided different particle size distributions, ending up in a different cluster. Also Barrera-Chamorro et al. (2025) concluded that calcium-pectate network formation could result in larger particle sizes within apple puree. We demonstrated that the balance between non-, medium-, and high-methylesterified regions within the pectin populations influenced the strength of calcium interactions, adding up to the complexity of correlating puree particle size distribution with pectin's structure.

The rheological parameters are primarily related to the sugar content of galactose, arabinose, and rhamnose in WSS, and arabinose and rhamnose in ChSS. These varying sugar contents significantly affect the abundance of branched structures of pectin, which may lead to different spatial conformations of the pectin. This in turn can impact the networking and water-binding capabilities of the pectin, ultimately influencing the viscosity and texture of the puree product.

Interestingly, the methyl-esters distribution parameters DB_abs_ and DB_PGme_ of WSS fractions, were found to correlate with the rheological parameters, while no such relationship was observed with only DM value of WSS pectin. The parameters DB_abs_ and DB_PGme_ represent the amounts of non- and low-methyl-esterified blocks in the pectin molecules, which can form cross-linking bonds with calcium or protein, to influence the viscosities of the puree even without stimulating gel formation. These results indicate that the rheological parameters of puree are more significantly affected by the distribution of non- and low-methyl-esterified blocks over the pectin molecule, rather than by the DM.

Cluster analysis was used to group the pectin characteristics based on their similarity (Fig. 3). Overall, the descriptive parameters for the methyl-ester distribution of both WSS and ChSS pectin, along with the sugar content in each pectin fraction, exhibited a high degree of proximity in their clustering. The clustering technique offers a way to organize and classify the data according to characteristics. It is interesting to observe that the descriptive parameters related to methyl ester distribution in both WSS and ChSS, as well as the sugar content of each pectin fraction, tended to cluster closely.

In general, as the results of PCA analysis and correlation analysis showed, the methyl-ester distribution parameters of WSS and ChSS pectin have an effect on the puree's texture. Previous studies have indicated that the texture of purees is influenced by particle size and quantity (Espinosa-Muñoz et al., 2013). The particle size would also further influence the grainy and crunchy mouthfeel of the puree product (Buergy, Rolland-Sabaté, Leca, & Renard, 2021). As the particle size distribution plot showed, the multimodal distribution may due to the presence of varying pectin structures and their interactions, leading to different aggregation behaviors and textural outcomes in the puree. Therefore, it can be inferred that the distribution of methyl-esterification in pectin may indirectly impact the texture of puree products based on our findings. The varied structural features, such as the distribution patterns of methyl esters in pectins, can influence the networks that trap water, leading to different water-holding capacities in plant-based liquid products like puree (Willats et al., 2006). Combining the information mentioned earlier, viscosity parameters (n-v and n-p) correlate with the methyl-esterification distribution parameters, especially those related to WSS, highlighting the complex regulation of puree rheology. These results confirm that pectin structural signatures collectively—rather than individually—determine puree properties, aligning with their varying textural outcomes. Moreover, the correlation between viscosity parameters and methyl ester distribution parameters suggest that the methyl-ester distribution of apple pectin may play an important role in puree quality, especially regarding texture.

## 4 Conclusion

The structural characterization of pectin from various apple cultivars reveals insights into its functional properties and implications for apple puree quality. Our findings indicate that the dry matter and pulp content significantly influenced puree physical properties like viscosity and colloidal stability, with rheological profiles confirmed shear-thinning behaviour and a broad consistency index range. We identified different roles for two distinct pectin fractions present in the apple: WSS pectins, characterized by high methyl-esterification, contribute to increased viscosity, while ChSS pectins exhibited various DM distributions and affected the particle size of the puree. Multivariate analyses established that methyl-ester distribution parameters had potential to predict the puree's quality index like particle size and viscoelastic profiles. Besides, despite some apple cultivars being related in the genetic lineage (e.g. Rui Xue & Rui Yang; Rui Xue & Pink Lady; Rui Yang & Fuji; Shen Fu & Fuji), the pectin structural characteristics varied among the different cultivars. Although there is a parentage relationship among related apple cultivars, no clear correlation between pectin structure and these cultivars was found, indicating that their genetic relationship does not necessarily predict pectin properties.

The interplay between pectin sugar composition and esterification plays a key role in rheological properties, with a balanced GalA content and uniform methyl-ester distribution affecting viscosity and stability. However, apple puree is a complex system in which multiple factors coordinate to influence its physical properties, making it difficult to predict its characteristics based on a single factor. Further research is needed to explore the mechanisms of their combined effects. Notably, the cultivar-dependent structural variations in pectin provide a valuable framework for predicting puree texture, suggesting that specific pectin profiles can be targeted for different applications in food processing. In addition, the presence and activity of apple endogenous enzymes, such as PG and pectin methyl esterase, might be crucial in fruit storage and processing, but still warrant further detailed research. This study bridges molecular-level insights with macroscopic puree behaviour, underscoring the potential for cultivar selection based on pectin structural characteristics to standardize product quality.

## CRediT authorship contribution statement

**Dazhi Liu:** Writing – original draft, Methodology, Investigation, Conceptualization. **Jinfeng Bi:** Supervision, Project administration, Funding acquisition. **Xuan Liu:** Writing – review & editing, Supervision. **Jianing Liu:** Conceptualization. **Henk.A. Schols:** Writing – review & editing, Supervision.

## Declaration of generative AI and AI-assisted technologies in the writing process

During the preparation of this work the authors used ChatGPT in order to check grammar. After using this tool, the authors reviewed and edited the content as needed and take full responsibility for the content of the published article.

## Declaration of competing interest

The authors declare that they have no known competing financial interests or personal relationships that could have appeared to influence the work reported in this paper.

## Data Availability

Data will be made available on request.

## Appendix A Supplementary data

Supplementary material Image 1
