# Supplementary material for: Apple cultivar-specific pectin properties impact apple puree functionality
Source: Food Chem X. 2025 Nov 13;32:103289. doi: 10.1016/j.fochx.2025.103289 (PMC12670112; doi:10.1016/j.fochx.2025.103289)
Supplement: Supplementary file 1 — Supplementary material [file mmc1.docx]

**Apple Cultivar-Specific Pectin Properties Impact Apple Puree Functionality**

Dazhi Liu^a,b^, Jinfeng Bi^a,^*, Xuan Liu^a, c^*, Jianing Liu^d^, Henk. A. Schols^b,^ *

*^a^ Institute of Food Science and Technology, Chinese Academy of Agricultural Sciences (CAAS), Key Laboratory of Agro-Products Processing, Ministry of Agriculture and Rural Affairs, Beijing 100193, China*

*^b^ Laboratory of Food Chemistry, Wageningen University & Research, Bornse Weilanden 9, 6708 WG, Wageningen, The Netherlands.*

*^c^ Institute of Western Agriculture, Chinese Academy of Agricultural Sciences, Changji, 831100, China*

*^d^ Department of Food Science and Nutrition, The Hong Kong Polytechnic University, Hong Kong SAR, China*

* Corresponding author.


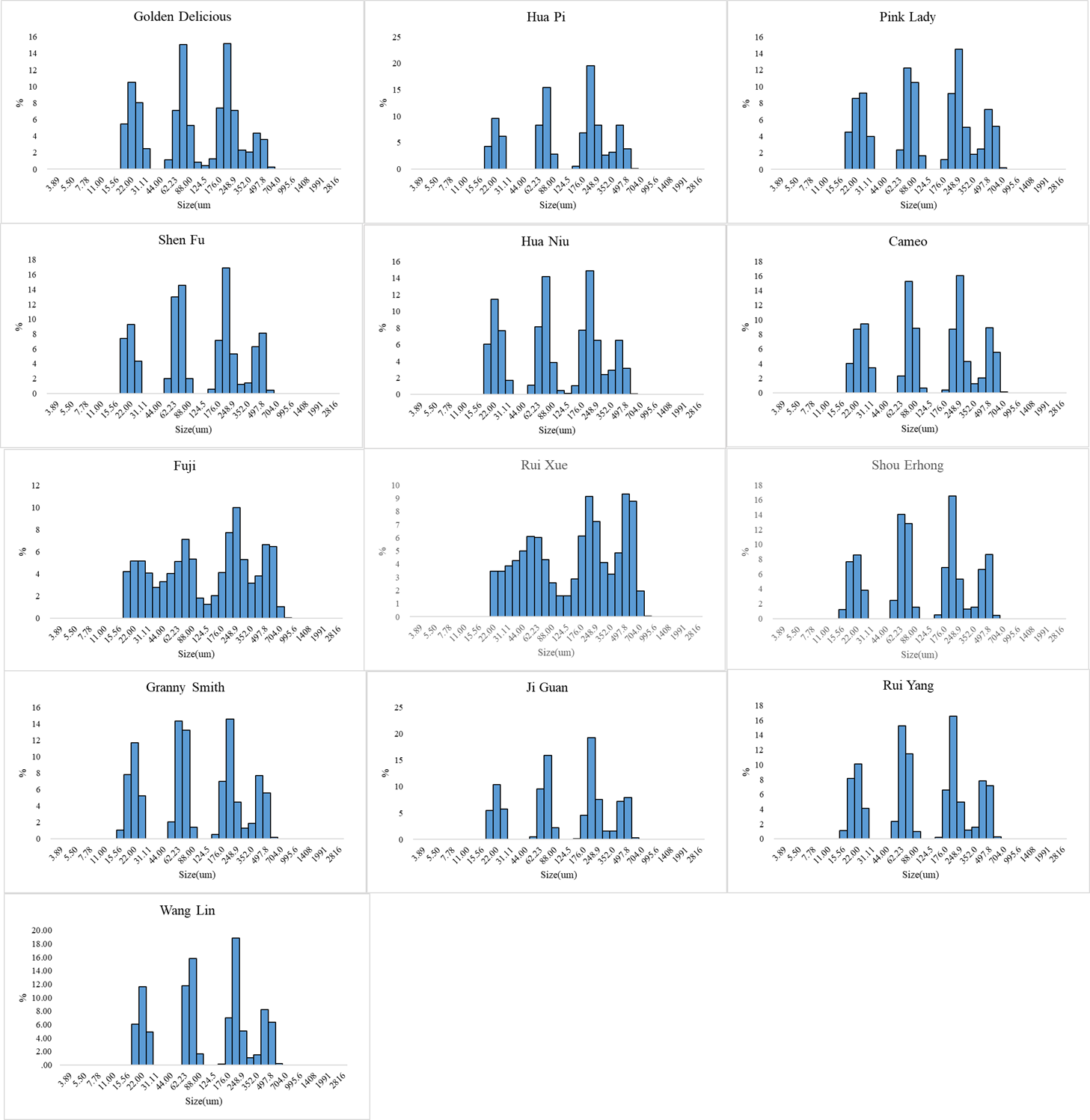


Fig. S1 Particle size distribution of apple puree from various apple cultivars.

Table S1 Sugar molar ratios of pectin fractions extracted from 13 different apple cultivar

|  | WSS | | | ChSS | | |
| --- | --- | --- | --- | --- | --- | --- |
|  | RG-I percentage (%) | HG to RG-I | Average side chain length | RG-I percentage (%) | HG to RG-I | Average side chain length |
| Golden Delicious | 20 | 17.8 | 8.5 | 21.2 | 14.1 | 5.9 |
| Hua Pi | 13.5 | 32.1 | 9.3 | 17.5 | 16.2 | 5.0 |
| Pink Lady | 18.1 | 16.1 | 7.1 | 12.1 | 33.4 | 7.3 |
| Shen Fu | 22.4 | 14.4 | 8.2 | 9.2 | 50.4 | 8.2 |
| Hua Niu | 13.6 | 26.6 | 7.1 | 8.6 | 45.2 | 6.6 |
| Cameo | 13.4 | 36.9 | 10.2 | 13.5 | 35.6 | 9.3 |
| Fuji | 18.2 | 27.8 | 11.0 | 9.1 | 45.2 | 7.1 |
| Rui Xue | 15.5 | 27.7 | 8.3 | 13.8 | 31.6 | 8.6 |
| Shou Erhong | 16 | 22.6 | 6.9 | 11 | 31.4 | 5.9 |
| Granny Smith | 29.6 | 19.2 | 17.7 | 15.3 | 60.4 | 19.9 |
| Ji Guan | 17.2 | 20.4 | 7.6 | 12.2 | 42.8 | 10.2 |
| Rui Yang | 31 | 12.3 | 12.1 | 10.9 | 49.5 | 10.1 |
| Wang Lin | 17.8 | 16.5 | 5.7 | 18 | 18.8 | 6.6 |
| **Average** | 18.9 | 22.4 | 9.2 | 13.3 | 36.5 | 8.5 |

RG-I percentage = (2*Rha+Ara+Gal)/100; HG to RG-I ratio = (GalA-Rha)/(2*Rha); Average RG-I side chain length = (Ara+Gal)/Rha
